# Gossypiboma, a Rare Cause of Abdominal Pain: A Case Report

**DOI:** 10.3390/reports8040242

**Published:** 2025-11-20

**Authors:** Doo Yong Son, Moon Han Choi

**Affiliations:** 1 Department of Family Medicine, Soonchunhyang University Gumi Hospital, Soonchunhyang University College of Medicine, Gumi 39371, Republic of Korea; s106162@schmc.ac.kr; 2 Department of Internal Medicine, Soonchunhyang University Gumi Hospital, Soonchunhyang University College of Medicine, Gumi 39371, Republic of Korea

**Keywords:** foreign bodies, foreign-body reaction, abdominal pain

## Abstract

Gossypiboma is a retained surgical item, most commonly gauze or sponge, inadvertently left inside a patient’s body after surgery. Although preventable, it can cause severe complications and is often underreported due to medicolegal concerns. We present a case of a 61-year-old woman who experienced left lower abdominal pain for three days. Her history included lumbar disc surgery via the lower left abdomen a decade earlier. Physical examination revealed a non-tender pelvic mass, and abdominal computed tomography (CT) showed a 4.5 × 4.7 × 6.1 cm high-attenuation lesion with internal low-attenuation areas in the left retroperitoneal space. The mass was surgically removed, and gauze material was identified inside, confirming the diagnosis of gossypiboma. The patient recovered uneventfully postoperatively. Gossypiboma can present with subacute or chronic symptoms, making diagnosis challenging. While uncommon, gossypiboma should be considered in differential diagnoses of patients with unexplained abdominal masses and prior surgical history. Prompt surgical management is essential to prevent complications. This case highlights the importance of meticulous surgical counts and awareness of this rare but serious condition.

**Figure 1:**
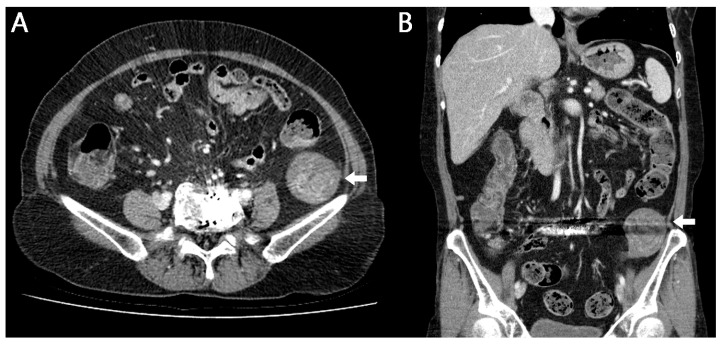
Retention of gauze, sponges, or various surgical instruments after surgery is rare [1]. Although this complication is entirely preventable, when it does occur, it may lead to severe, potentially life-threatening consequences. Nevertheless, the true incidence is likely underreported because of medicolegal concerns [2]. Gossypiboma, a term combining gossypium (Latin for cotton) and boma (Swahili for “to hide”), refers specifically to retained surgical textiles [3]. Gossypiboma is an uncommon yet significant postoperative complication resulting from retained surgical materials. Despite its rarity, it can present with a wide range of clinical manifestations and may lead to substantial morbidity. Its incidence, clinical features, and preventive strategies have been extensively discussed in the literature. Here, we report a case of gossypiboma discovered on abdominal computed tomography (CT) in a patient who presented to our hospital with abdominal pain. The diagnosis was confirmed following surgical resection of the abdominal mass. A 61-year-old female patient visited our hospital complaining of pain in the left lower abdomen that began 3 days ago. The patient said that she had a history of lumbar disc surgery at another hospital 10 years ago and the surgery was performed on the lower left abdomen. The patient had undergone lumbar disc surgery at another hospital, and although the exact surgical approach was not documented, the procedure was presumed to have been performed via a retroperitoneal approach based on imaging findings and the operative scar location. In addition, there was a history of coil embolization for subarachnoid hemorrhage 4 years ago. The physical examination revealed blood pressure 120/70 mmHg, pulse rate 68/min, respiration rate 20/min, and body temperature 36.5 °C and the patient showed acute illness. On abdominal examination, a relatively immobile mass without tenderness was palpable in the left pelvic region. Peripheral blood test showed white blood cell 6390/mm^3^ (Neutrophil 60.9%), hemoglobin 12.3 g/dL, and platelet 205,000/mm^3^. Biochemistry analysis showed aspartate aminotransferase (AST)/alanine aminotransferase (ALT) 17/12 IU/L, total/direct bilirubin 0.5/0.2 mg/dL, blood urea nitrogen 18 mg/dL, creatinine 0.5 mg/dL, and C-reactive protein (CRP) 0.5 mg/dL. Abdominal CT image: (**A**) axial and (**B**) coronal CT images demonstrate metal artifacts from previous lumbar disc surgery and an ovoid 4.5 × 4.7 × 6.1 cm sized poorly enhancing low-attenuation lesion with inner whirled high-attenuation portions in retroperitoneal space of left upper pelvic cavity (arrow).

**Figure 2:**
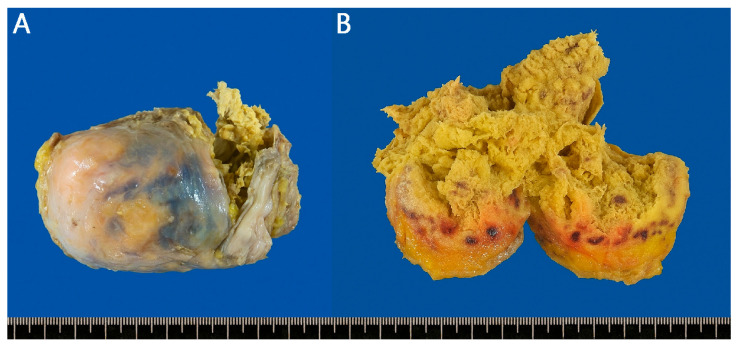
The patient was referred to a surgeon, and the following day, a Laparoscopic surgery was performed via a transabdominal approach to access the retroperitoneal space. A round, tightly encapsulated mass measuring 5 × 5 × 8 cm was resected from the retroperitoneal side of the sigmoid colon. Gross findings revealed a chronic inflammatory reaction with necrotic tissue and retained gauze within the lesion (Figure 2). Ultimately, the patient was diagnosed with gossypiboma resulting from a foreign body reaction to gauze left behind during a previous surgery. The patient was discharged on postoperative day 8 without complications, and no recurrent symptoms were observed during subsequent short-term follow-up. Postoperative gross findings: (**A**) A round, tightly encapsulated mass measuring 5 × 5 × 8 cm was resected from the retroperitoneal side of the sigmoid colon. (**B**) Incision revealed a chronic inflammatory reaction with necrotic tissue and gauze inside. Gossypiboma often presents with subacute or chronic symptoms that differ from typical cases of acute abdominal pain [4]. Because diagnosis is challenging and associated with significant medicolegal concerns, reporting rates are low, making it difficult to accurately estimate its true incidence. Although the number of cases in Korea remains unclear, a 2003 report from the United States estimated an incidence of 1 case per 8801 to 18,760 surgeries [5], and a 2015 report documented an incidence of 1.32 cases per 10,000 surgeries [6]. Factors significantly associated with increased risk included emergency surgery, unplanned surgical changes, and body mass index, as well as changes in intraoperative nursing staff and increased blood loss. The abdomen and pelvis are the most common sites of occurrence, although cases have also been reported in the vagina, thoracic cavity, and other locations [5]. The clinical presentation of gossypiboma varies depending on the location of the retained material and the type of tissue reaction involved [7]. Gossypiboma results from gauze, sponges, or other surgical materials inadvertently left in the body after surgery. Two distinct types of foreign body reactions are described pathologically. The first type is an exudative reaction, which can lead to serious complications such as abscess formation, internal or external fistula development, intestinal adhesion, obstruction, or perforation, potentially progressing to sepsis and even death. This type typically produces more severe and acute symptoms [8]. The second type, as observed in the present case, is an aseptic fibrotic reaction characterized by adhesion, encapsulation, and ultimately granuloma formation. This process may remain asymptomatic for many years, and lesions are often detected incidentally [9]. In this case, the granuloma likely developed through an aseptic fibrotic reaction, given the patient’s history of lumbar disc surgery 10 years prior and the absence of other symptoms. The mass appears to have formed without intestinal adhesion, obstruction, or perforation, likely because the retained gauze was located in the retroperitoneal space of the left upper pelvic cavity. For diagnosis, simple X-ray image may suggest gossypiboma if radiopaque markers are visible [10]. On ultrasonography, it typically appears as a well-circumscribed mass with a hypoechoic rim and strong posterior acoustic shadowing, often containing wavy internal echogenic foci [11]. CT findings vary, but the most characteristic features include a sponge-like mass with internal air bubbles or a well-defined round lesion with marked peripheral wall enhancement [12]. Because gossypiboma is uncommon, often asymptomatic, and difficult to detect, accurate preoperative diagnosis remains challenging even when imaging studies are performed. It must be differentiated from other causes of abdominal pain, such as mesenteric ischemia, appendicitis, cholecystitis, diverticulitis, tumors or recurrences presenting with intestinal obstruction or intra-abdominal masses, and intra-abdominal abscesses. If gossypiboma is suspected, timely surgical intervention is essential. Therefore, in patients presenting with abdominal pain who have relevant risk factors and a history of prior surgery, or when radiopaque non-transmissive markers are detected on plain radiography, the possibility of gossypiboma—such as in the present case—should be strongly considered.

## Data Availability

The data presented in this study are available on request from the corresponding author (the data are not publicly available due to they contain information that could compromise patient privacy).

## Abbreviations

The following abbreviations are used in this manuscript:

CT	Computed Tomography
